# The double-edged sword effect of argumentative scaffolding on group discussion in an adaptive discussion system

**DOI:** 10.3389/fpsyg.2022.997522

**Published:** 2022-11-10

**Authors:** Hongli Gao, Sheng Xu, Lei Yang, Xiangen Hu

**Affiliations:** ^1^School of Psychology, Xinxiang Medical University, Xinxiang, Henan, China; ^2^School of Psychology, Central China Normal University, Wuhan, Hubei, China; ^3^School of Psychology, Henan Normal University, Xinxiang, Henan, China

**Keywords:** artificial intelligence in education, intelligent tutoring system, group discussion, cognitive diversity, argumentation, argumentative knowledge construction

## Abstract

Group discussion is a common and important form of learning. The effectiveness of group discussion could be facilitated by the adaptive support of virtual agent. Argumentative knowledge construction is beneficial to learners’ acquisition of knowledge, but the effectiveness of argumentative scaffolding is not consistent in existing studies. In this study, a total of 64 college students (32 groups, two participants and one computer agent in each group) participated in the experiment and they were assigned to the experimental condition (16 groups) and the control condition (16 groups). In the control condition, the computer agent would give an idea from semantically different categories according to the automatic categorization of the current discussion. In the experimental condition, the computer agent provided argumentative scaffolding after giving diverse ideas to support participants’ deep processing. The argumentative scaffolding included two prompt questions, “do you agree with me?” and “could you give the reasons to support your viewpoint?.” The dependent variables were the interaction quality, network centrality, the breadth and depth of discussion, the self-reported of discussion effectiveness and the degree of change before and after the discussion. Findings revealed that compared with the control condition, the participants were more likely to discuss the keywords provided by the virtual agent and reported more comprehensive understanding of the discussion topic, but surveyed less ideas and interactions during the discussion under the argumentative condition. This study suggests that the argumentative scaffolding may have both positive and negative effect on the group discussion and it’s necessary to make a choice.

## Introduction

Group discussion is a regular activity in learning and even in life. The generation and innovation of knowledge are realized in the interaction between old and new knowledge during the discussion. The collaborative knowledge construction involved in the discussions plays an important role in recent learning theories, such as social constructivism, connectionism, and new constructivism. However, the effect of discussion is unwarranted without scaffolding. As we experience in our daily lives, not all discussions are equally effective. Even in the same discussion group, each member’s discussion effect is different. The primary factor that makes group discussions effective is whether the new information emerges during the discussion. In the same group, all members receive the same information, but the results of participating in the discussion are not the same. One of the important reasons may lie in whether they conduct deep cognitive processing on the new information they receive. Therefore, the factors influencing the effect of group discussion and its mechanism need to be explored.

Idea generation is an important cognitive processing activity in group discussion. According to the search for ideas in associative memory (SIAM) model (Nijstad and Stroebe, 2006) and cognitive-social-motivational (CSM) model (Paulus and Brown, 2007; Paulus and Kenworthy, 2021) in group idea generation, ideas from others will serve as cognitive stimulation which activates the less accessible knowledge especially when these stimuli are semantically diverse. Therefore, cognitive diversity and further processing of these diverse information are the important features of productive discussion.

Breadth and depth of discussion are important metrics to reflect the effectiveness of group discussion. Breadth of discussion was measured by the number of categories, while the depth of discussion was measured by the number of ideas per category (Nijstad et al., 2002). Because it was hard to manipulate the content of ideas in real interactive groups, Nijstad et al. (2002) used an idea exposure paradigm instead and found that compared to a control condition, participants generated more diverse ideas (increased breadth) in the diverse stimuli condition and generated more ideas per category (increased depth) in the homogeneous stimuli condition. Baruah and Paulus (2016) found that highly related (homogeneous) categories yielded higher within-category fluency (increased depth) than low related categories. In previous studies, the manipulation of cognitive diversity was established before group discussion, which would not dynamically change with the current semantic domain in the discussion process and was a static difference without adaptability. However, an important aspect of group performance lied in the internal dynamic process under discussion, and the real impact of static cognitive differences on group activities was manifested in the dynamic process. Gao et al. (2019) proposed the theoretical and operational definitions of adaptive cognitive diversity. In an intelligent discussion system, the computer agent provided dynamic support for group discussion. Results showed that compared to the homogeneous condition, the breadth of discussion was increased but the depth of discussion was not decreased under the diverse condition. Previous studies showed that semantically diverse ideas increased the range of accessible knowledge and allowed for the improvement of breadth of production, but the impact of these diverse ideas on depth of discussion were inconsistent (Nijstad et al., 2002; Baruah and Paulus, 2016; Gao et al., 2019).

Deep discussion means that learners work together to acquire knowledge by exchanging views and arguments, negotiating meaning, and (co-)constructing knowledge, which is also called argumentative knowledge construction. There is a range of mechanisms to explain the beneficial impact of argumentation on learning. Firstly, developing arguments may involve the elaboration of the content in which relations between prior knowledge and new information may be employed. Secondly, providing arguments in argumentative discussion may require learners to make more explicit the inferential relations between various pieces of information. Furthermore, exchanging arguments may spark off conceptual change and thereby deepen learners’ understanding of complex ideas (Wecker and Fischer, 2014). Wolfe et al. (2018) argue that an argument is, minimally, a claim supported by a reason. However, learners may have difficulties in constructing well-grounded arguments, rarely build upon the arguments of their learning partners and their arguments may lack important components (Stegmann et al., 2007; Weinberger et al., 2007). Collaborative argumentation-based learning (CABLE) has been used to facilitate peer collaboration and knowledge construction. Computer supported collaborative learning (CSCL) recently has been seen as an important and achievable instructional strategy to facilitate and support CABLE for deeper understanding and providing productive arguments. Meta-analysis revealed that further research on the design of collaborative argumentation software is clearly required (Scheuer et al., 2010). Research on the effectiveness of argumentative scaffolding has shown divergent results, argumentative scaffolding was successful for enhancing argumentation, but the mean effect of the interventions on domain specific knowledge seemed to be non-existent (Wecker and Fischer, 2014; Noroozi et al., 2017; Vogel et al., 2017).

The scripts are particularly effective when they prompt transactive activities and when they are combined with content-specific scaffolding that are designed to support the processing of content-related information (Vogel et al., 2017). Ertl et al. (2008) focused on the effects of supporting learners with external representations (textually represented collaboration scripts and graphically represented content schemes) during collaborative case-solving, and the results showed that learners particularly benefited from the graphically represented content scheme. Vogel et al. (2022) assumed that granting learners the opportunity to adapt the collaboration scripts and heuristic worked examples scaffolds to their self-perceived needs might be a way to further enhance their effects in solving mathematical conjecture problems, and results showed that adaptable CSCL scripts were partly helpful for students with higher levels of self-regulation skills. Although there are similarities between these studies, direct comparisons are difficult because they differ in aspects such as learning tasks, instructional support, measures of learning outcomes and so on.

To sum up, there has been a lot of research on collaborative learning, idea generation and argumentative knowledge construction. However, group discussion has similarities with them but also has its own characteristic and there is not much research on the dynamic cognitive mechanism in group discussion. We can draw on the results of other related fields to explore the issues in the group discussion.

Providing dynamic adaptive support for online discussion by using artificial intelligence technology (e.g., natural language processing) is a promising but underdeveloped field. Adaptive collaborative learning support, where computer-supported collaborative learning (CSCL) and artificial intelligence in education (AIED) research intersect, has recently received increasing attention (Rummel et al., 2016). Intelligent support for learning in groups was a common topic at recent AIED and intelligent tutoring system (ITS) conferences. Among all kinds of adaptive collaborative learning support systems, conversational agent-based support system is a common type. At the interpersonal level, pedagogical agents could scaffold learning by giving task-related messages, prompts, and hints during the learning process (Sikström et al., 2022).

In order to broaden a topic, learners need to look at the topic from different perspectives and aspects to clarify different subtopics, while in order to deepen a topic, learners need to elaborate their ideas in depth by using reasonable evidence and examples (Noroozi, 2021). The present study combined dynamic content-related scaffolding with content-independent scaffolding to prompt transactive activities to improve the effectiveness of group discussion. In the adaptive discussion system, the virtual agent can automatically recognize the type of the subject’s current discussion content and conduct adaptive dialogue with the subjects. In present study, after the automatic categorization of participants’ current contributions, the computer agent provided diverse ideas (content-specific scaffolding) and asked about attitudes and reasons for these ideas (content-independent scaffolding) to facilitate participants’ further processing of content-related information in the experimental condition. In the control condition, the computer agent only provided diverse ideas (content-specific scaffolding). Besides the breadth and depth of discussion, more metrics were used to reflect the effectiveness of discussion, for example, network centrality, interaction quality, self-reported discussion effectiveness and so on. We hypothesize that the depth of discussion (quality of interaction, the number of views per category) would be better but the breadth (the number of categories, the number of stating opinions) of discussion could be worse in the experimental condition.

## Materials and methods

### Participants and task

A total of 64 undergraduates (9 boys and 55 girls) participated in this study, which consisted of 32 discussion groups in pairs. During the experiment, they generated ideas at a computer terminal on the topic of “how will artificial intelligence affect humans?” and would be paid a small amount of money after the experiment. The participants provided their written informed consent to participate in this study. Informed consent included the main content of this study, confidentiality, freedom of withdrawal and so on.

### Design and materials

Experiment design. Participants were randomly assigned to the experimental condition (16 groups) and the control condition (16 groups), and participants who knew each other were divided into different discussion groups. In the control condition, the computer agent would give an idea from semantically different categories according to the automatic categorization of the current discussion. In the experimental condition, after giving diverse ideas, the computer agent asked about the participants’ attitudes and reasons for these ideas to support participants’ deep processing.

Corpus. The typical answers to discussion question “how will artificial intelligence affect humans?” were collected and grouped into eight common categories (Gao et al., 2019). This study selected one typical viewpoint from each type as the corpus for computer agent (see Table 1).

**Table 1:** The view library of virtual agent.

Type	Typical view
Convenience	I think artificial intelligence will make people’s life more convenient.
Unemployment	The popularity of artificial intelligence will still make some people unemployed.
Dependency	People rely too much on artificial intelligence, some abilities will gradually lose.
Control	We should legislate as soon as possible and have detailed management and constraints on artificial intelligence in various industries.
Progress	Emancipate people from simple labor, so that they can devote more energy to better intelligent creation.
Reasonable use	Reasonable use will make human life more beautiful, nonrational use will cause harm.
Trend	I think the development of artificial intelligence is an irresistible trend.

Argumentative scaffolding. Because the reason or basis for supporting the opinion is an indispensable part of the argument, the argumentative scaffolding in this study included two prompt questions, “do you agree with me?” and “could you give the reasons to support your viewpoint?”

Pre-test and post-test questionnaires (see Appendix). The pre-test questions include whether you have taken relevant courses, how interested you are in the topics discussed, and how many times you have viewed them in the past week. The post-test questionnaire was scored from the aspects of whether the discussion process fully expressed their own views, the degree of help from others’ views, the overall discussion effect, and the willingness to further discuss. There was an essay question “how will artificial intelligence affect humans?” in the pre-and post-test questionnaires to measure participants’ understanding of the problem before and after the discussion. In order to lighten the burden of the subjects, the question was answered by recording.

### Adaptive discussion system

This study was conducted in an adaptive discussion system (Gao et al., 2019), and all the experimental procedures including pre-test questionnaire, online discussion, post-test questionnaire were completed on this platform. During the online discussion stage, the computer agent was displayed in the corresponding area with the code “GX07” as ID, and participants could communicate with the virtual agent synchronously. The virtual agent can automatically identify the category of participants’ current contributions mainly by keyword matching and conduct an adaptive dialogue with the subject. The accuracy of computer classification was evaluated. It turned out that the consistency (the number of consistent marks divided by the number of total marks) between two researchers was 0.72, and the consistency between computer and manual marking was 0.79.

### Procedure

The whole experiment process was conducted in a laboratory with a partition. Before the experiment, the experimenter set up a discussion group in the management interface to determine the discussion group number and member ID.

Pre-test stage. In this stage, participants were asked to answer a subjective question “how will artificial intelligence affect humans?.” In order to lighten the burden of the participants, the question was answered by recording. With the function of speech-to-text of Sougou, the speech can be converted into text in real time, and then the experimenter proofread and modify the transferred text against the recording. After the recording, participants were taken to the computer to begin the formal experiment. There were partitions around each seat to avoid interference with each other. Different subjects in the same group were arranged in different positions, and they were not placed in opposite or adjacent positions. Then the computer screen showed the general instruction interface to welcome the arrival of the subjects, introduce the experimental procedures and confidentiality principle, guide the subjects to fill in the pre-test questionnaire.

Online discussion stage. After submitting the pre-test questionnaire, participants entered the online discussion stage. The instruction in this section introduced the topic and time of online discussion, and informed participants that GX07 was a computer agent. The group discussion would not begin until all three members join. During the discussion, the topic of “how will artificial intelligence affect humans? Let us start the discussion!” was displayed on the top of the discussion interface. In the experimental condition, when participants provided a total of 3–7 valid ideas or 7 invalid ideas continuously or no one spoke for more than a minute, the virtual agent provided feedback and the discussion process entered argumentation mode. The feedback provided by the virtual agent includes responses (50% probability), different types of opinions, and questions about their attitudes and reasons. In the argumentation mode, if the participants provided a valid opinion, they would be given a favorable response (50% probability). If no one spoke for 1 min, the virtual agent encouraged them to speak more. If they digressed five times in a row or no one spoke for more than 2 min, the system forced him or her out of the argumentation mode. During the whole discussion, the same type of opinion will appear at most once. After eight types of views were presented, the virtual agent stopped talking. In the control condition, the computer agent would give an idea from semantically different categories according to the automatic categorization of the current discussion but would not provide the argumentative scaffolding to ask the attitude and reason of the participants, nor did it provide the prompt to encourage them to speak more.

Post-test stage. After 25 min, the discussion stage was terminated and entered the post-test questionnaire interface. After submitting the questionnaire, the participants went into another room for a post-test recording. At the end of the post-test recording, participants were asked to judge the depth and breadth of their understanding of the topic discussed and to make a choice. Then, the depth and breadth of the pre-test were judged by comparing the text transferred from the pre-test recording. In the end, the participants were paid.

### Dependent variables

The network centrality. The main purpose of this experiment was to reveal the influence of the argumentative scaffolding provided by the virtual agent on the discussion process. According to previous study (Oshima et al., 2012), the key words in the perspective provided by the virtual agent were taken as nodes, and the co-occurrence of key words or synonyms were taken as the relationship between nodes. The network centrality was the number of other points in the network that were directly connected to one point. The higher the network centrality of a point was, the more important it was in the network.

The interaction quality. In this study, the development of the analysis framework was a theoretical and data-driven spiral process. The coding framework was based on the Interaction Analysis Model (IAM) proposed by Gunawardena et al. (1997). Firstly, the existing analysis framework was used to analyze the text of the discussion process. In the process of data analysis, the coding framework was constantly revised to better reveal the impact of the debate framework. For example, in the process of coding, it was found that the interaction behavior of “modification and supplement to the original viewpoint” was quite common, and the corresponding type could not be found in the original IAM system. Therefore, this type was added in the stage of “sharing and comparing information,” which is called “modification.” The final coding framework included two phases: sharing/comparing of information (statement of opinion, statement of agreement, providing evidence, asking and answering, modification), discovery and exploration of dissonance or inconsistency (statement of disagreement, views from a different category, providing evidence, asking and answering, modification).

This study was coded by two researchers familiar with the topic. The coding process included the following stages: Coding exercises. Similar experimental data were selected for coding exercises. The practice phase began by coding independently, then put the results together to negotiate differences and reach a consensus. Then, new experimental data were selected for independent coding and inconsistent negotiation. The researchers need to do three rounds of the exercises. The coding unit. A message send by the subject was finally determined to be the coding unit. If multiple types were involved in the same message, multiple types would be encoded. Formal coding process. During the formal coding process, the same batch of experimental data was encoded by two researchers. First, they coded independently, then calculated the consistency, negotiated the differences and reached a consensus. Based on the results of negotiation between them, the number of occurrences of each type in each discussion group was calculated. The final data analysis was mainly to reveal the influence of the views provided by the virtual agent on the discussion process, thus, only the words said by the subjects after the virtual agent’s speech was counted, the sum of the times of each type of the two subjects was taken as the overall indicator of the subjects.

The breadth and depth of discussion. Each sentence in the chat log was classified by the computer. Sentences that did not involve any of the keywords in the list was marked as invalid views, and valid views are marked as the corresponding type. On the basis of this classification, the number of views and categories of subjects 1 and 2 in each group can be calculated. In this study, two subjects in the same group were analyzed as a whole. The total number of views was equal to the number of subject 1’s views plus the number of subject 2’s views. The total number of categories was equal to the sum of the two subjects’ categories minus the number of repeated categories. On this basis, the following dependent variables were analyzed: The breadth of discussion was the number of categories surveyed by participants in the discussion. The depth of discussion was the number of views per category (equal to the number of views divided by the number of types; Nijstad et al., 2002). The ratio of valid ideas was equaled to the number of valid ideas divided by the number of total sentences.

The self-reported discussion effectiveness. The self-reported of discussion effectiveness mainly from three aspects through the post-test questionnaire, including helpfulness from others’ point of view, overall discussion effectiveness and willingness to further discussion.

The degree of change before and after the discussion. The computer marked the number of types involved in the transformed text of the recording, and on this basis, we calculated the total increase of the number of types tested before and after the discussion. The total increase was also calculated for the scores of the depth and breadth self-assessment questionnaires.

## Results

### Comparison of the interaction quality between the two groups

The interaction quality was calculated by the coding results of the two researchers. After several rounds of independent coding, consistency calculation and negotiation of inconsistencies, the coding consistency of the two researchers was above 0.8.

The independent-samples T test was conducted for each type of sentence under two conditions. The results showed that for the statement of opinion, the experimental group (*M* = 3.13) was significantly lower than that of the control group (*M* = 6.88), *t*(30) = −3.65, *p* < 0.01, *d* = −1.29. In terms of questions and answers in stage 1, the experimental group (*M* = 3.31) was significantly lower than the control group (*M* = 8.00), *t*(30) = −2.37, *p* < 0.05, *d* = −0.84. In other indicators, there was no significant difference between the two conditions (see Table 2).

**Table 2:** Comparison of the interaction quality between the two conditions.

Phase	Category	Experimental group (*n* = 16) *M* ± *SD*	Control group (*n* = 16) *M* ± *SD*	*t*	*d*
	Unrelated	7.50 ± 9.95	18.81 ± 30.60	−1.14	
Sharing/Comparing of information	Opinion	3.13 ± 1.89	6.88 ± 3.65	−3.65**	−1.29
	Agreement	6.69 ± 3.96	6.44 ± 4.07	0.18	
	Evidence	3.94 ± 2.17	4.00 ± 3.39	−0.06	
	Question and answer	3.31 ± 4.45	8.00 ± 6.54	−2.37*	−0.84
	Modification	11.75 ± 6.42	14.25 ± 9.13	−0.90	
Discovery and exploration of dissonance or inconsistency	Disagreement	0.13 ± 0.34	0.13 ± 0.34	0.00	
	Views from a different category	1.81 ± 1.22	1.56 ± 1.86	0.45	
	Evidence	0.19 ± 0.40	0.13 ± 0.34	0.47	
	Question and answer	0.13 ± 0.34	0.06 ± 0.25	0.59	
	Modification	1.75 ± 2.35	0.69 ± 1.35	1.57	

*Means *p* < 0.05; **Means *p* < 0.01.

### Comparison of the network centrality between the two groups

**Figure 1:**
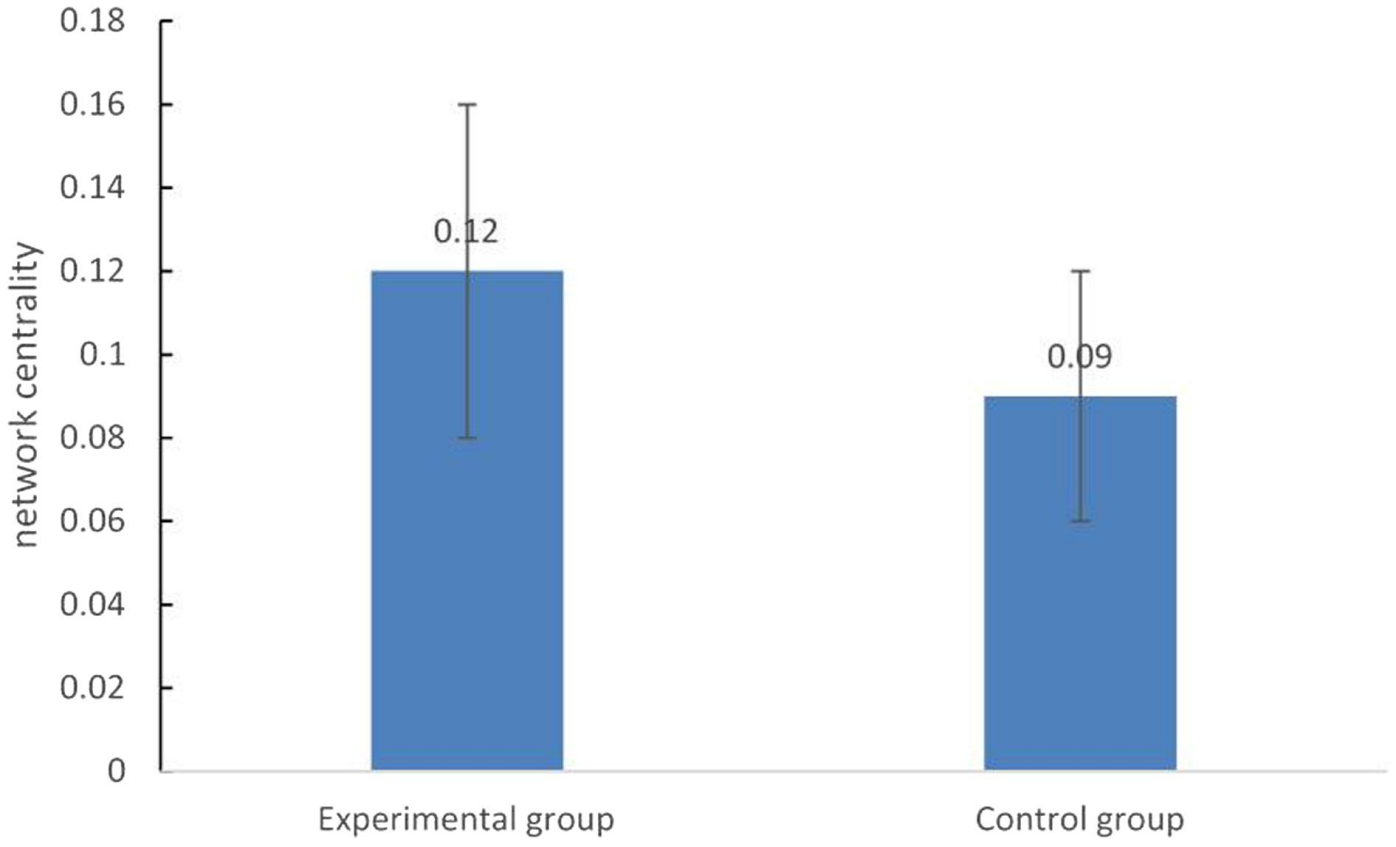
Comparison of the network centrality between the two groups.

Taken the viewpoint first proposed by the virtual agent as nodes, the association between nodes was represented by the co-occurrence of keywords, the degree of centrality of keywords in the network was calculated, and the difference between the two conditions was compared. The results showed that the degree centrality (*M* = 0.12) of the key words provided by the virtual agent under the experimental condition was significantly higher than that under the control condition (*M* = 0.09), *t*(30) =2.35, *p* < 0.05, *d* = 0.85 (see Figure 1).

### Comparison of the breadth and depth of discussion between the two groups

The independent-samples T test was conducted for the number of types, views and sentences, the average number of views per category and percentage of valid views under the two conditions. The results showed that in terms of the proportion of effective viewpoints, the experimental group (*M* = 0.7) was significantly higher than the control group (*M* = 0.57), *t*(30) =2.22, *p* < 0.05, and *d* = 0.79. In other indicators, there was no significant difference between the two groups (see Table 3).

**Table 3:** Comparison of the breadth and depth of discussion between the two conditions.

	Experimental group (*n* = 16) *M* ± *SD*	Control group (*n* = 16) *M* ± *SD*	*t*	*d*
Number of types	7.06 ± 0.68	7.31 ± 0.79	−0.96	
Number of views	27.75 ± 9.23	34.31 ± 13.71	−1.59	
Number of sentences	44.38 ± 24.53	67.81 ± 40.03	−2.00	
Average number of views per category	3.90 ± 1.15	4.61 ± 1.55	−1.48	
Percentage of valid views	0.70 ± 0.17	0.57 ± 0.16	2.22*	0.79

*Means *p* < 0.05.

### Comparison of the self-reported discussion effectiveness between the two groups

**Figure 2:**
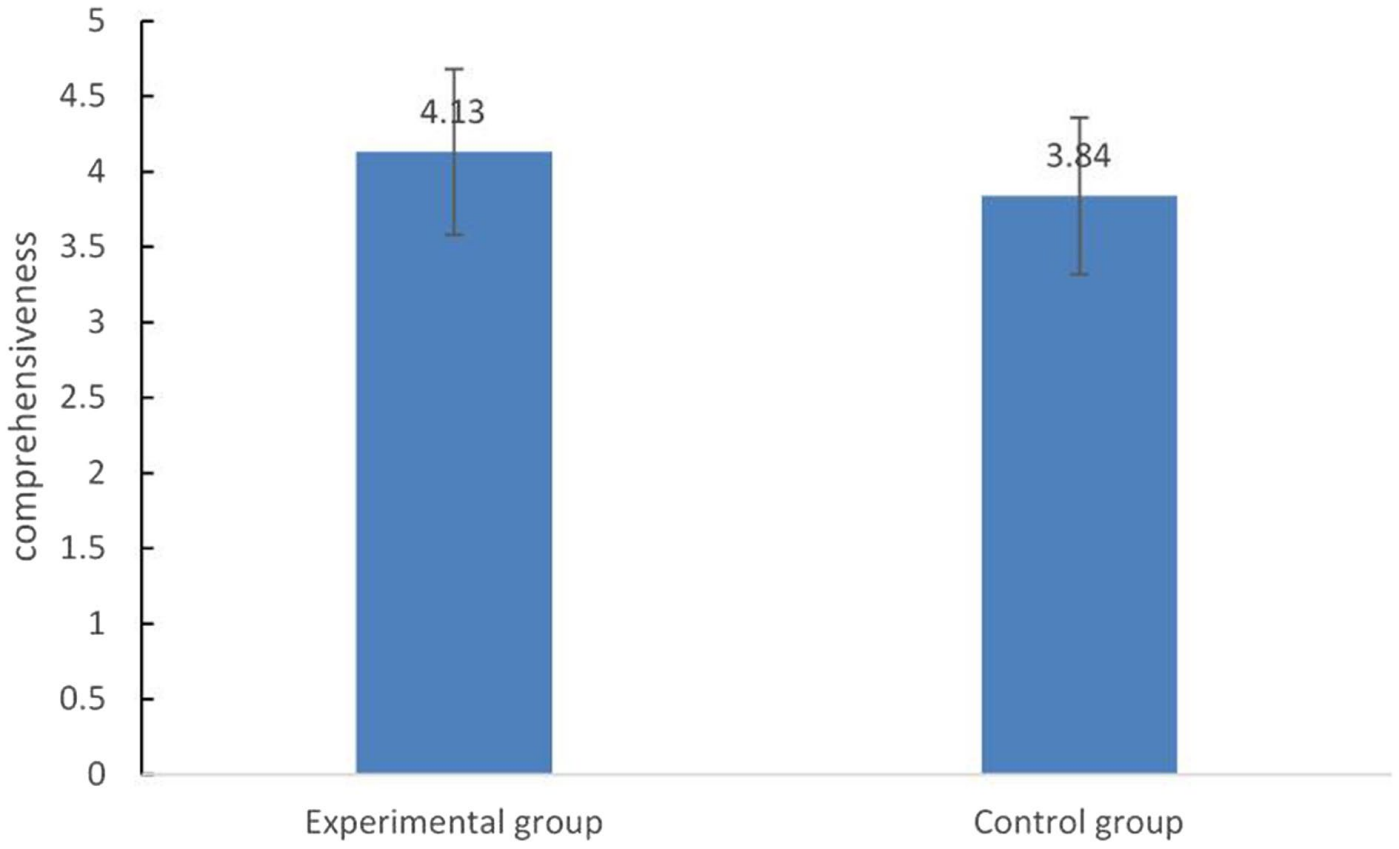
Comparison of the self-reported discussion effectiveness between the two groups.

The independent-samples T test was carried out on the pretest level of the groups under the two conditions, and the results showed that there was no significant difference. The differences between the two conditions were compared in the self-assessment scores in the post-test questionnaire on the helpfulness of others’ opinions, the effect of discussion and the intention to discuss again. The results showed that in terms of the comprehensive understanding of the topic, the experimental group (*M* = 4.13) was significantly higher than the control group (*M* = 3.84), *t*(62) =2.10, *p* < 0.05, *d* = 0.54. On the other items, there were no significant differences (see Figure 2).

### Comparison of the degree of change between the two groups

The independent-samples T test was conducted on the degree of change before and after the discussion. The results showed that there was no significant difference between the two conditions.

## Discussion

Group discussion is an independent processing activity which overlaps with collaborative learning, group idea generation and argumentative knowledge construction. Theories from those similar fields could be used to explore the rules of group discussion. In this study, the effect of dynamic content specific scaffolding and content independent scaffolding on small group discussion was investigated in an adaptive discussion system and more indexes were used to measure the effectiveness of discussion (e.g., interaction quality, the percentage of valid views, self-reported discussion effectiveness, the degree of change). In the experimental condition, after providing a semantically diverse view (content specific scaffolding) which is from a different category from participants’ previous contributions, the virtual agent would ask the participants if they agree with the view and to give their reasons (content independent scaffolding). The results illustrated that compared with the control condition, the participants would follow what the computer agent said and reported more comprehensive understanding of the discussion topic, but surveyed less view statements, questions and answers in the experimental condition. These results suggested that argumentative scaffolding had both positive and negative influence on group discussion.

### Effect of argumentative scaffolding on depth of discussion

This study focused on the effect of argumentative scaffolding on the depth of discussion. In order to deepen a topic, learners need to elaborate their ideas in depth by using reasonable evidence and examples (Noroozi, 2021). In present study, the purpose of inquiring attitude was to make the subjects think whether they agree or disagree with the different views and provide an opportunity to argue. The purpose of inquiring reasons was to make the subjects provide evidence to support the views and construct the views in depth. The degree of debate and construction is an important indicator to evaluate the depth of interaction (Gunawardena et al., 1997). The result of interaction quality analysis showed that there was no significant difference in expressing disagreement and providing evidence between the two conditions, which meant that the content-independent scaffolding in the experimental condition did not provoke more debates. Furthermore, the number of expressing agreement was much greater than the number of expressing disagreement, which might be because the viewpoint base of the virtual agent in this study was made up of common viewpoints, which was less likely to cause debates.

Another indicator to reflect depth of discussion is the average number of views per category (Nijstad et al., 2002; Baruah and Paulus, 2016; Gao et al., 2019). The average number of views per category was computed by dividing the number of views by category. In the experimental condition, the computer agent asked about the attitude towards the idea provided by the agent and the reasons to support the attitude. The result of present study showed that there was no significant difference in the depth of discussion between the experimental condition and the control condition, which was inconsistent with the expected assumption. This is probably because the participants spent more time answering the questions posed by the computer agent rather than coming up with new ideas, so that there was no significant difference between the number of views and categories. According to the calculation method of depth of discussion (the number of views divided by the number of categories), it is understandable that there was no significant difference in the depth of discussion.

### Positive effect of argumentative scaffolding

The results of a meta-analysis by Vogel et al. (2017) suggested that domain specific knowledge acquisition would be more beneficial when collaborative scripts prompt transactive activities and when they are combined with additional content specific scaffolding. In order to broaden a topic, learners need to look at the topic from different perspectives and aspects to clarify different subtopics (Noroozi, 2021). At the highest level, CSCL scripts may be combined with domain-specific scaffolds that are designed to support the processing of content-related information in problem solving tasks. According to the transactivity principle in the script theory of guidance, Fischer et al. (2013) postulated that “the more a given CSCL practice requires the transactive application of knowledge, the better this knowledge is learned through participation in this CSCL practice.” In present study, after the automatic categorization of participants’ current contributions, the computer agent provided diverse ideas (content-specific scaffolding) and then asked about the attitudes and reasons for these ideas (content-independent scaffolding) to facilitate participants’ further processing of content-related information. We supposed that the synergistic scaffolding (combining the content specific scaffolding with content independent scaffolding) could prompt transactive activities (i.e., builds upon or directly refers to a contribution of a learning partner). The results of the network analysis with the key words provided by the virtual agent as nodes showed that the degree centrality was higher in the argumentative condition which meant that participants were more likely to discuss the keywords provided by the virtual agent (Oshima et al., 2012). The proportion of valid viewpoints of the subjects in the discussion also confirmed this point. Under the experimental condition, the subjects were discussing around the topic, and there were few words irrelevant to the topic. From the point of view of the self-evaluation index of discussion effectiveness, the subjects subjectively felt that they had a more comprehensive understanding of the problem under the experimental condition. These results illustrated the positive effect of argumentative scaffolding.

### Negative effect of argumentative scaffolding

Vogel et al. (2022) found that adaptable scaffolding may be a too high burden on the learners and has only limited benefits when compared to non-adaptable scaffolding. The review by Noroozi et al. (2012) suggests that highly structured interventions may result in the side effects of argumentative knowledge construction. This hypothesis was supported by the interaction acts in present study. The number of stating an opinion, questioning and answering participants surveyed during the discussion was lower in the experimental condition compared with the control condition. When subjects spend more time discussing problems raised by virtual agents, they have less time to propose new ideas in the limited time range. These results reflect the negative effect of argumentative scaffolding.

However, there was no significant difference in the number of expressing attitude and providing evidence under the two conditions, which was not consistent with what we expected. After further analysis of the chat logs, it was found that subjects preferred to illustrate their views by giving examples which were marked as providing evidence in both of the conditions, so there was no significant difference in the number of providing evidence under the two conditions. Furthermore, the subjects would express agreement or disagreement both after the virtual agent and after another subject in the same group offering an opinion. Because of the presence of the second subject, the role of the virtual agent was weakened, so that there was no significant difference in the number of sentences of expressing attitude.

### Limitations and future directions

So far, we have tried to collect the answers for the subjective questions before and after the discussion with free answers, keywords, text and recording, etc., but they do not reflect the differences at different levels of the independent variable. The self-evaluation of the depth and breadth of the eight types in this study did not reflect the differences under different experimental conditions as well. This may be because it is difficult to sensitively reflect the changes before and after the discussion. Just as in everyday discussions, we can feel that our understanding of the problem under discussion has changed, but it is difficult to express what has changed. For open discussion, it is difficult to express the discussion effect directly in a short time. In the future, we should consider using indirect approaches, such as performance on similar tasks, as an indicator of the transfer of discussion effects.

The corpus used by the virtual agent was the common views combed from the previous discussion records, to which the participants generally agreed. In the future, we should consider to supplement the viewpoints that may cause controversy so as to trigger further argumentative knowledge construction. Furthermore, the automatic classification was limited to eight predetermined types and future studies can give adaptive feedback based on semantic similarity and explore its impact on the discussion effect. In addition, the self-improving mechanism of virtual agents should be considered to be included in the future research, so the original corpus could be supplemented and improved with the progress of the discussion process (Long et al., 2020).

## Conclusion

This study explored the dynamic cognitive process in group discussion, i.e., the effect of content related scaffolding and content independent scaffolding on the effectiveness of discussion. The results illustrated that argumentative scaffolding played a double-edged role in group discussion. On the one hand, the scaffolding facilitated the participants to focus the discussion on the current topic and to have a more comprehensive understanding of the problem. On the other hand, when participants spent more time on the views provided by the computer agent, they would not have enough time to come up with new ideas and interact with others.

These findings suggested that in the case of limited discussion time, depth and breadth of discussion are contradictory. We should weigh the gains and losses and make a decision according to the purpose of the discussion. If the purpose of the discussion is to get different perspectives, it is more effective for the computer to provide only different types of perspectives. If the purpose of the discussion is to deepen the understanding of the current point of view, the computer can provide an argumentative scaffolding to guide the subject to further processing.

## Data availability statement

The raw data supporting the conclusions of this article will be made available by the authors, without undue reservation.

## Ethics statement

Ethical review and approval was not required for the study on human participants in accordance with the local legislation and institutional requirements. The patients/participants provided their written informed consent to participate in this study.

## Author contributions

HG contributed to the study design, data collecting, and paper writing. SX contributed to the construction of intelligent discussion system and automatic data analyzing. LY contributed to data collecting and analyzing. XH guided the whole study. All authors contributed to the article and approved the submitted version.

## Funding

This research was funded by the grant to the first author HG from Humanities and Social Science Research Project of Ministry of Education “The Influence of Semantic Similarity Based Adaptive Cognitive Diversity on Discussion Effect” (20YJC880016) and General Project of Education Science Planning in Henan Province “Research on the Construction of Intelligent Case Discussion System Based on Cognitive Diversity” (2021YB0163). Any opinions, findings, and conclusions or recommendations expressed in this material are those of the authors and do not necessarily reflect the views of Ministry of Education of the People’s Republic of China.

## Conflict of interest

The authors declare that the research was conducted in the absence of any commercial or financial relationships that could be construed as a potential conflict of interest.

## Publisher’s note

All claims expressed in this article are solely those of the authors and do not necessarily represent those of their affiliated organizations, or those of the publisher, the editors and the reviewers. Any product that may be evaluated in this article, or claim that may be made by its manufacturer, is not guaranteed or endorsed by the publisher.

## Appendix

**Pre-test questionnaire**

1. I have taken courses related to artificial intelligence.

**Table:**

(1) Yes	(2) No

2. I’m interested in artificial intelligence.

**Table:**

(1) Strongly agree	(2) Agree	(3) Neutral	(4) Disagree	(5) Strongly disagree

3. I always pay attention to information related to artificial intelligence.

**Table:**

(1) Strongly agree	(2) Agree	(3) Neutral	(4) Disagree	(5) Strongly disagree

4. How many times have you read about artificial intelligence in the past week?

**Table:**

(1) More than three times	(2) Three times	(3) Twice	(4) Once	(5) Zero

**Post-test questionnaire**

1. I fully expressed my views during the discussion.

**Table:**

(1) Strongly agree	(2) Agree	(3) Neutral	(4) Disagree	(5) Strongly disagree

2. During the discussion, the opinions of others are very helpful to me.

**Table:**

(1) Strongly agree	(2) Agree	(3) Neutral	(4) Disagree	(5) Strongly disagree

3. What Computer GX07 said gave me a different perspective.

**Table:**

(1) Strongly agree	(2) Agree	(3) Neutral	(4) Disagree	(5) Strongly disagree

4. After discussion, I have a more comprehensive understanding of the question “how will artificial intelligence affect human beings.”

**Table:**

(1) Strongly agree	(2) Agree	(3) Neutral	(4) Disagree	(5) Strongly disagree

5. After discussion, I have a deeper understanding of the question “how will artificial intelligence affect human beings.”

**Table:**

(1) Strongly agree	(2) Agree	(3) Neutral	(4) Disagree	(5) Strongly disagree

6. I think this discussion has worked well.

**Table:**

(1) Strongly agree	(2) Agree	(3) Neutral	(4) Disagree	(5) Strongly disagree

7. At the end of the discussion, I felt that I had not said what I wanted to say.

**Table:**

(1) Strongly agree	(2) Agree	(3) Neutral	(4) Disagree	(5) Strongly disagree

8. I’ll have this discussion again.

**Table:**

(1) Strongly agree	(2) Agree	(3) Neutral	(4) Disagree	(5) Strongly disagree
